# Standardized Uptake Values Derived from ^18^F-FDG PET May Predict Lung Cancer Microvessel Density and Expression of KI 67, VEGF, and HIF-1*α* but Not Expression of Cyclin D1, PCNA, EGFR, PD L1, and p53

**DOI:** 10.1155/2018/9257929

**Published:** 2018-06-06

**Authors:** Alexey Surov, Hans Jonas Meyer, Andreas Wienke

**Affiliations:** ^1^Department of Diagnostic and Interventional Radiology, University of Leipzig, Leipzig, Germany; ^2^Institute of Medical Epidemiology, Biostatistics, and Informatics, Martin-Luther-University Halle-Wittenberg, Halle, Germany

## Abstract

**Background:**

Our purpose was to provide data regarding relationships between ^18^F-FDG PET and histopathological parameters in lung cancer.

**Methods:**

MEDLINE library was screened for associations between PET parameters and histopathological features in lung cancer up to December 2017. Only papers containing correlation coefficients between PET parameters and histopathological findings were acquired for the analysis. Overall, 40 publications were identified.

**Results:**

Associations between SUV and KI 67 were reported in 23 studies (1362 patients). The pooled correlation coefficient was 0.44. In 2 studies (180 patients), relationships between SUV and expression of cyclin D1 were analyzed (pooled correlation coefficient = 0.05). Correlation between SUV and HIF-1*α* was investigated in 3 studies (288 patients), and the pooled correlation coefficient was 0.42. In 5 studies (310 patients), associations between SUV and MVD were investigated (pooled correlation coefficient = 0.54). In 6 studies (305 patients), relationships between SUV and p53 were analyzed (pooled correlation coefficient = 0.30). In 6 studies (415 patients), associations between SUV and VEGF expression were investigated (pooled correlation coefficient = 0.44). In 5 studies (202 patients), associations between SUV and PCNA were investigated (pooled correlation coefficient = 0.32). In 3 studies (718 patients), associations between SUV and expression of PD L1 were analyzed (pooled correlation coefficient = 0.36). Finally, in 5 studies (409 patients), associations between SUV and EGFR were investigated (pooled correlation coefficient = 0.38).

**Conclusion:**

SUV may predict microvessel density and expression of VEGF, KI 67, and HIF-1*α* in lung cancer.

## 1. Introduction

Lung cancer is one of the most frequent malignancies in humans [1]. It is the largest cause of cancer deaths in the United States [1].

Multiple histopathological factors influence tumor biology in lung cancer. According to the literature, different molecular markers play a key role here [2]. Previous reports investigated numerous biomarkers and suggested that some histopathological parameters can predict tumor behavior in lung cancer [2–5]. It has been shown that they provide information about tumor proliferation, aggressiveness, prognosis, and therapy response [2–5]. According to the literature, following biomarkers are relevant in lung cancer: proliferation index KI 67, hypoxia-inducible factor- (HIF-) 1*α*, tumor suppressor protein p53, vascular endothelial growth factor (VEGF), epidermal growth factor receptor (EGFR), proliferating cell nuclear antigen (PCNA), PD L1, and several cyclins [2–10]. For instance, it has been shown that tumors with high expression of KI 67 and/or VEGF were associated with a worse prognosis [3, 4]. Similar results were also reported for expression of HIF-1*α* and p53 [5–7].

Furthermore, some reports analyzed associations between imaging parameters and histopathological features in lung cancer [11–14]. Especially parameters of positron emission tomography (PET) like standardized uptake values (SUV) were in focus of the studies. However, the reported data were inconsistent. While some authors found such significant relationships, others did not. Therefore, it is unclear whether SUV can be used as a surrogate parameter reflecting histopathological features in lung cancer or not.

The purpose of this meta-analysis was to provide evident data about associations between SUV and histopathological parameters in lung cancer.

## 2. Materials and Methods

### 2.1. Data Acquisition

**Figure 1:**
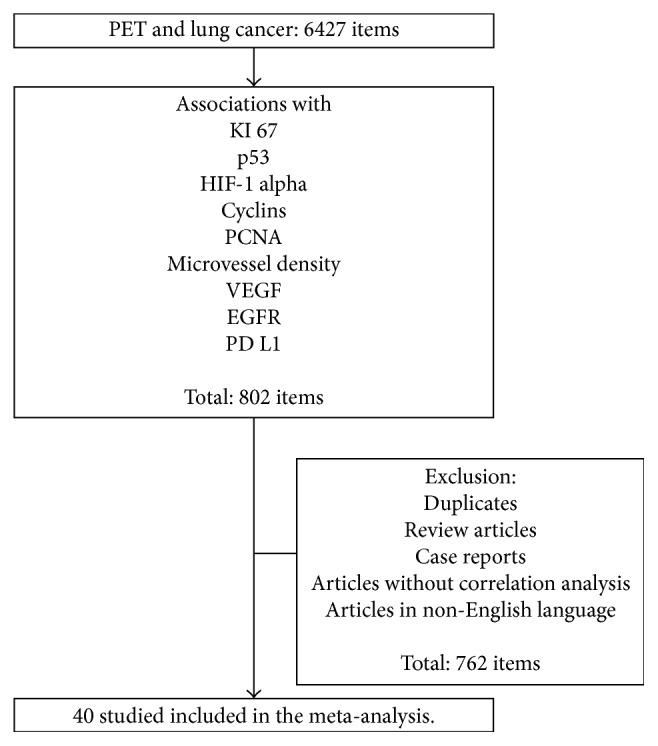
Flowchart of the data acquisition.

The strategy of data acquisition is shown in Figure 1. MEDLINE library was screened for associations between PET parameters and histopathological findings in lung cancer up to December 2017.

For associations between PET and different biomarkers, the following search words were used:

- *PET and KI 67*: “lung cancer AND PET OR positron emission tomography AND KI 67 OR KI67 OR ki67 OR ki-67 OR mitotic index OR proliferation index OR MIB 1 OR MIB-1 OR mitosis index” (192 items)
- *PET and expression of p53*: “lung cancer AND PET or positron emission tomography AND p53 OR tumor suppressor protein” (51 items)
- *PET and expression of VEGF*: “lung cancer AND PET or positron emission tomography AND VEGF OR vascular endothelial growth factor” (82 items)
- *PET and expression of EGFR*: “lung cancer AND PET or positron emission tomography AND EGFR OR epidermal growth factor receptor” (345 items)
- *PET and expression of HIF-1α*: “lung cancer AND PET or positron emission tomography AND HIF-1*α* OR HIF1*α* OR HIF-1 alpha OR HIF1 alpha OR hypoxia-inducible factor” (38 items)
- *PET and expression of PCNA*: “lung cancer AND PET or positron emission tomography AND PCNA OR proliferating cell nuclear antigen” (23 items)
- *PET and expression of cyclins*: “lung cancer AND PET or positron emission tomography AND cyclin” (22 items)
- *PET and microvessel density*: “lung cancer AND PET or positron emission tomography AND microvessel density OR MVD” (34 items)
- *PET and expression of PD L1*: “lung cancer AND PET or positron emission tomography AND programmed cell death-ligand 1 OR PD L1” (15 items).

Secondary references were also recruited. Overall, 802 records were identified. After exclusion of doublets, review articles, case reports, non-English publications, and articles, which not contain correlation coefficients between PET and histopathological parameters, there were 40 articles [11–50].

The following data were extracted from the literature: authors, year of publication, number of patients, histopathological parameters, and correlation coefficients, according to our previous descriptions [51–53].

The Preferred Reporting Items for Systematic Reviews and Meta-Analyses statement (PRISMA) was used for the research [54].

### 2.2. Meta-Analysis

The methodological quality of the acquired 40 studies was independently checked by two observers (Alexey Surov and Hans Jonas Meyer) using the Quality Assessment of Diagnostic Studies (QUADAS) instrument according to previous descriptions [55].  Table 1 shows the results of QUADAS proving.

Associations between PET and histopathological findings were analyzed by Spearman's correlation coefficient. The reported Pearson's correlation coefficients in some studies were converted into Spearman's correlation coefficients according to the previous description [56].

Furthermore, the meta-analysis was undertaken by using RevMan 5.3 (Computer Program, version 5.3, The Cochrane Collaboration, 2014, The Nordic Cochrane Centre, Copenhagen). Heterogeneity was calculated by means of the inconsistency index *I*^2^ [57, 58]. Additionally, DerSimonian and Laird random-effects models with inverse-variance weights were used without any further correction [59].

## 3. Results

### 3.1. KI 67

**Figure 2:**
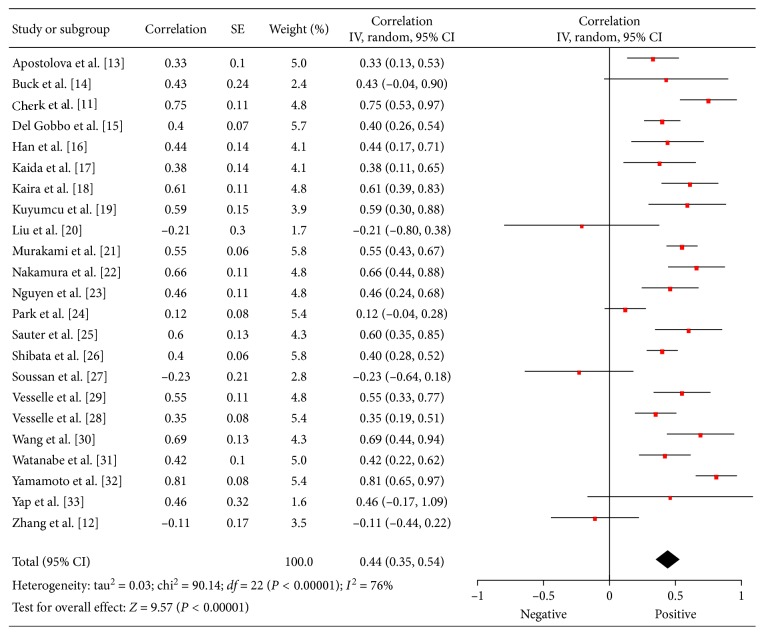
Forest plots of correlation coefficients between SUV_max_ and KI 67 in patients with lung cancer.

Associations between ^18^F-FDG PET and KI 67 were reported in 23 studies (1362 patients) [11–33]. The calculated correlation coefficients between SUV_max_ and KI 67 ranged from −0.23 to 0.81 (Figure 2). The pooled correlation coefficient was 0.44 (95% CI = (0.35; 0.54)).

### 3.2. Cyclin D1

**Figure 3:**
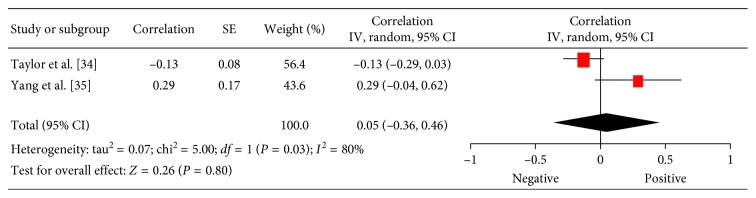
Forest plots of correlation coefficients between SUV_max_ and expression of cyclin D1.

In 2 studies (180 patients), relationships between 18F-FDG PET and expression of cyclin D1 were analyzed [34, 35]. The pooled correlation coefficient between these parameters was 0.05 (95% CI = (−0.36; 0.46)) (Figure 3).

### 3.3. HIF-1*α*

**Figure 4:**
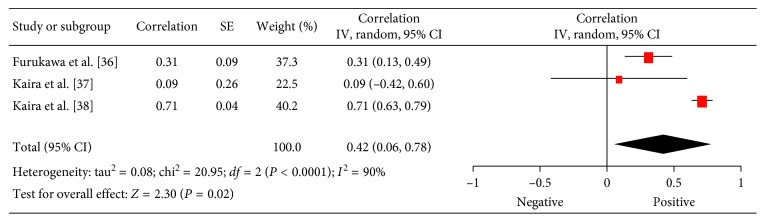
Forest plots of correlation coefficients between SUV_max_ and expression of HIF-1*α* in lung cancer.

Associations between ^18^F-FDG PET and HIF-1*α* were investigated in 3 studies (288 patients) [36–38]. The reported correlation coefficients ranged from −0.19 to 0.99 (Figure 4). The pooled correlation coefficient was 0.42 (95% CI = (0.06; 0.78)).

### 3.4. Microvessel Density (MVD)

**Figure 5:**
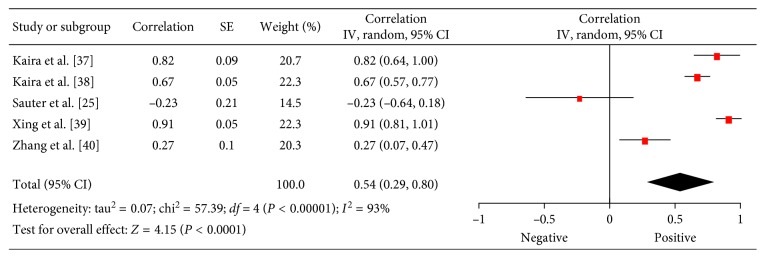
Forest plots of correlation coefficients between SUV_max_ and microvessel density.

Associations between ^18^F-FDG PET and MVD were investigated in 5 studies (310 patients) [25,37–40]. The reported correlation coefficients ranged from −0.23 to 0.91 (Figure 5). The pooled correlation coefficient was 0.54 (95% CI = (0.29; 0.80)).

### 3.5. P53

**Figure 6:**
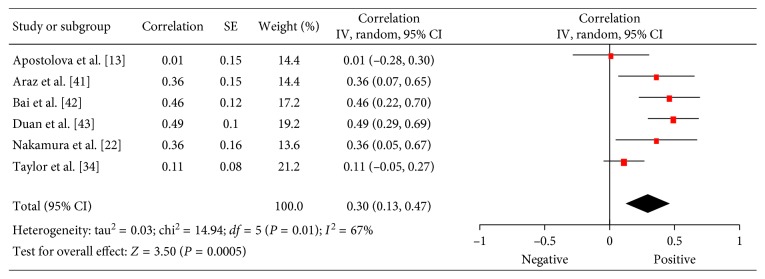
Forest plots of correlation coefficients between SUV_max_ and expression of p53.

In 6 studies (305 patients), relationships between ^18^F-FDG PET and p53 were analyzed [13,22,34,41–43]. The pooled correlation coefficient between these parameters was 0.30 (95% CI = (0.13; 0.47)) (Figure 6).

### 3.6. VEGF

**Figure 7:**
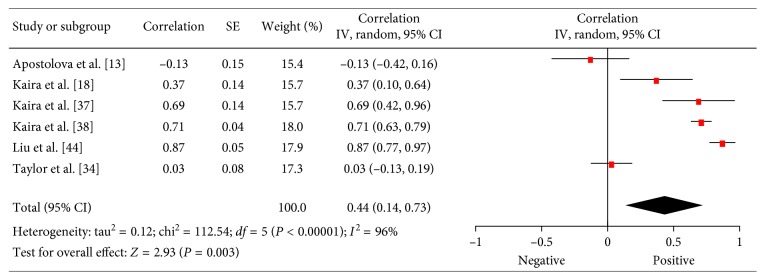
Forest plots of correlation coefficients between SUV_max_ and VEGF expression.

There were 6 studies (415 patients) which investigated associations between SUV and expression of VEGF in lung cancer [13, 18, 34, 37, 38, 44]. The reported correlation coefficients ranged from −0.13 to 0.77 (Figure 7). The pooled correlation coefficient was 0.44 (95% CI = (0.14; 0.73)).

### 3.7. PCNA

**Figure 8:**
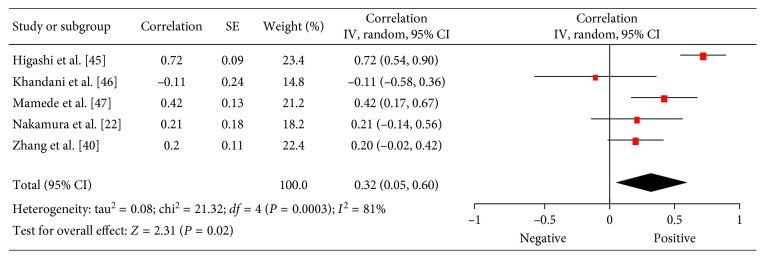
Forest plots of correlation coefficients between SUV_max_ and PCNA.

There were 5 studies (202 patients) which investigated associations between ^18^F-FDG PET and PCNA in lung cancer [22,40,45–47]. The reported correlation coefficients ranged from 0.04 to 0.83 (Figure 8). The pooled correlation coefficient was 0.32 (95% CI = (0.05; 0.60)).

### 3.8. EGFR

**Figure 9:**
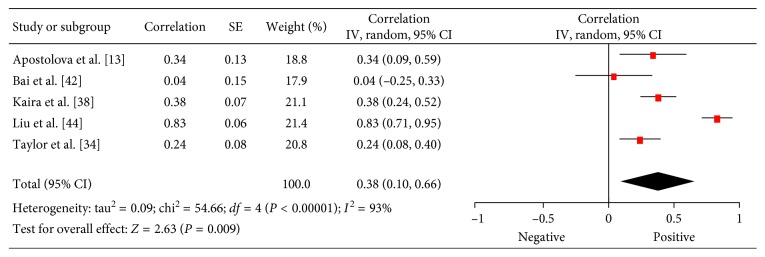
Forest plots of correlation coefficients between SUV_max_ and EGFR expression.

There were 5 studies (409 patients) which investigated associations between ^18^F-FDG PET and expression of EGFR in lung cancer [13, 34, 38, 42, 44]. The reported correlation coefficients ranged from 0.04 to 0.83 (Figure 9). The pooled correlation coefficient was 0.38 (95% CI = (0.10; 0.66)).

### 3.9. PD L1

**Figure 10:**
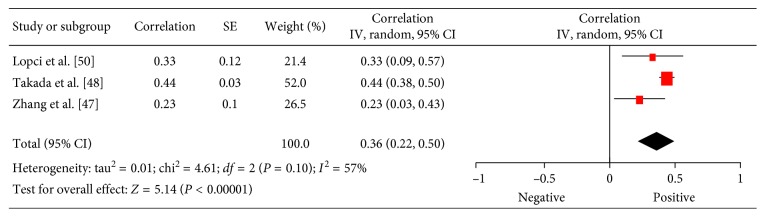
Forest plots of correlation coefficients between SUV_max_ and EGFR expression.

In 3 studies (718 patients), relationships between ^18^F-FDG PET and expression of PD L1 were analyzed [48–50]. The pooled correlation coefficient between these parameters was 0.36 (95% CI = (0.22; 0.50)) (Figure 10).

## 4. Discussion

Analysis of interactions between imaging findings, in particular, between PET and histopathology can significantly improve oncologic diagnostics [60]. The possibility to characterize histological tissues by imaging can also personalize anticancer treatment [60]. Although PET is an established investigation of lung cancer in clinical practice, only few reports analyzed the question if there are relationships between PET findings and different histopathological parameters. However, this is a key question. In fact, if PET parameters do correlate with several histopathological findings reflecting proliferation or other features of lung cancer, then PET values can also be used as biomarkers.

Our meta-analysis showed that SUV can reflect different histopathological parameters in lung cancer. As shown, SUV correlated moderately with KI 67. This finding is not surprisingly. KI 67 is a nonhistone, nuclear protein synthesized throughout the whole cell cycle except the G0 phase and has been shown to be responsible for cell proliferation [61]. It is an established biomarker in lung cancer for prediction of tumor behavior. Our data are in agreement with those of previous investigations and also analyzed relationships between expression of KI 67 and SUV in lung cancer [62, 63]. However, we found weak correlations between SUV_max_ and other proliferation markers, namely, PCNA (0.32). This finding is difficult to explain. Theoretically, SUV reflects metabolic activity and, therefore, might correlate stronger with several proliferation biomarkers. Obviously, metabolic activity and proliferation are not associated directly.

Similarly, our analysis found only slight correlation between SUV_max_ and expression of EGFR (0.38). EGFR is a cell membrane tyrosine kinase receptor [64, 65]. As reported previously, EGFR signaling is critical in development and cellular homeostasis, proliferation, and growth [64–66]. EGFR is overexpressed in most lung cancers [64–66]. Overexpression of EGFR is associated with a poor prognosis in non-small-cell lung cancer [66]. In addition, EGFR overexpression is also associated with chemoresistance in non-small-cell lung cancer [64, 66]. The present meta-analysis showed that SUV_max_ cannot be used as a surrogate marker for EGFR expression in lung cancer.

Furthermore, we analyzed associations between SUV_max_ and expression of p53. As seen, these parameters correlate weakly (0.30). According to the literature, p53 is a protein encoded by the TP53 gene and plays a key role in tumor suppression and in the cellular response to DNA damage [2, 5]. Some authors indicated that high expression of p53 can be used as a predictor for better overall survival [2]. However, in the study of Tsao et al., p53 protein overexpression was a significant prognostic marker of shortened survival [5]. Relationships between SUV_max_ and p53 were analyzed in 6 previous studies with divergent results [13,22,34,41–43]. Our data suggest that SUV cannot be used as a surrogate marker for expression of p53.

Programmed cell death-ligand 1 or PD L1 is another very important biomarker in lung cancer [67]. PD L1 is an immune modulator that promotes immunosuppression by binding to PD-1 receptor [68]. PD L1 on the surface of tumor cells inhibits an immune-mediated attack by binding to PD-1 on cytotoxic T-cells [68, 69]. According to the literature, high expression of PD L1 is associated with shorter overall survival in patients with non-small cell lung cancer [70]. Therefore, prediction of PD L1 expression by imaging may be of interest in clinical practice. Our analysis identified only a slightly correlation (0.36) between SUV_max_ and PD L1 expression in lung cancer; that is, SUV_max_ cannot be used as a surrogate marker for PD L1 status.

Our analysis also showed that SUV_max_ cannot predict expression of cyclin D1 in lung cancer. As reported previously, data of the role of this protein are inconsequent. For example, Gautschi et al. found a strong pathological role for cyclin D1 deregulation in bronchial neoplasia [71]. However, Zhang et al. suggested in their meta-analysis that the expression of cyclin D1 is unlikely to be useful as a prognostic marker for NSCLC in clinical practice from current evidence [72].

The present meta-analysis identified a moderate pooled correlation between SUV_max_ and hypoxia-inducible factor-1 alpha (HIF-1*α*). According to the literature, HIF-1*α* characterizes cellular responses to hypoxic stress [6, 7]. It has been reported that patients with lung cancer and positive HIF-1*α* expression in tumor tissues had lower overall survival rate than patients with negative HIF-1*α* expression [6, 7]. Furthermore, in a recent meta-analysis, it was suggested that HIF-1*α* expression may be a prognostic biomarker for lung cancer [6]. In addition, it is discussed that HIF-1*α* might be a target for therapy in lung cancer [7]. Therefore, associations between PET parameters and HIF-1*α* may be also of clinical importance.

Similarly, we calculated a moderate pooled correlation between SUV_max_ and expression of VEGF. Previous reports indicated that VEGF overexpression is associated with poor prognosis for NSCLC patients [3]. Furthermore, VEGF plays an important role in sustaining the development and progression of lung cancer [73]. Notably, some reports indicated a great potential of anti-VEGF agents in therapy of lung cancer [74]. Therefore, possible relationships between VEGF expression and SUV in lung cancer may play a significant role to plane chemotherapy. In fact, if SUV or other PET parameters may predict VEGF expression and tumors with overexpression, respectively, then PET may also be used for therapy control with anti-VEGF agents.

Finally, the strongest correlation was found between SUV_max_ and microvessel density (0.54). This finding seems to be logical. In fact, high metabolic activity may induce a high perfusion, which is associated with more vessels. SUV may identify hypervascularized tumor areas. Therefore, SUV may be used for evaluation of response to therapy with angiogenesis inhibitors.

The present meta-analysis also identified several other problems. Overall, most analyzed biomarkers are associated with SUV. This finding suggests that SUV_max_ may reflect different histopathological features in lung cancer. However, as mentioned above, the calculated pooled correlations are slightly-to-moderate. Therefore, our analysis showed that SUV_max_ cannot be used as an ultimate one-to-one surrogate marker for different receptor expressions in lung cancer.

Some reports suggested that other PET parameters like metabolic tumor volume or total lesion glycolysis are more sensitive than SUV_max_ [75]. In fact, pretreatment SUV is commonly used as a relative measure of ^18^FDG uptake and is considered a prognostic factor for risk stratification in different malignancies. However, as suggested previously, it does not reflect the heterogeneity of a tumor [76]. Therefore, to overcome this drawback of SUV, other PET parameters, such as metabolic tumor volume and total lesion glycolysis that reflect metabolic volume and activity, have been proposed as quantitative indexes of tumor metabolism [76, 77]. According to the literature, these parameters can be used as prognostic factors for survival in several malignant diseases like non-small lung cancer, pleural mesothelioma, and ovarian cancer [77–79]. Clearly, further researches are needed to investigate possible associations between several PET parameters and histopathology in lung cancer.

Furthermore, lung cancer involves several carcinomas with different histopathological features and behavior. Presumably, different subtypes of lung cancer may have also different associations between PET and histopathology. This question should also be analyzed by further investigations.

There were also other problems. Only 40 reports with small number of patients investigated associations between PET parameters and histopathological features in lung cancer. Furthermore, most of the acquired studies were retrospective. Finally, according the QUADAS criteria, all involved studies showed partial verification bias, differential verification bias, and incorporation bias. Also, most of the studies had clinical review bias and diagnostic review bias. Clearly, further prospective studies with more patients are needed to investigate associations between PET and histopathology in lung cancer.

Some recent reports indicated that other histopathological markers like tumor-infiltrating CD8-positive T lymphocytes, cyclooxygenase-2, and survivin play also a great role in lung cancer [3, 4]. However, there were either no data or in each case only one report about relationships between PET and these histopathological factors. This should be also the purpose for further investigations.

In conclusion, our meta-analysis showed that SUV_max_ may predict microvessel density and expression of VEGF, KI 67, and HIF-1*α* in lung cancer. There were no significant associations between SUV_max_ and expression of cyclin D1, EGFR, PD L1, PCNA, and p53.

## Figures and Tables

**Table 1:** Methodological quality of the involved 40 studies according to the QUADAS criteria.

QUADAS criteria	Yes (%)	No (%)	Unclear (%)
Patient spectrum	38 (95.0)	—	2 (5.0)
Selection criteria	28 (70.0)	1 (2.50)	11 (27.5)
Reference standard	40 (100)	—	—
Disease progression bias	40 (100)	—	—
Partial verification bias	40 (100)	—	—
Differential verification bias	40 (100)	—	—
Incorporation bias	40 (100)	—	—
Text details	40 (100)	—	—
Reference standard details	40 (100)	—	—
Text review details	16 (40.0)	4 (10.0)	20 (50.0)
Diagnostic review bias	17 (42.5)	4 (10.0)	19 (47.5)
Clinical review bias	39 (97.5)	—	1 (2.5)
Uninterpretable results	39 (97.5)	—	1 (2.5)
Withdrawal explained	38 (95.0)	1 (2.5)	1 (2.5)

## Data Availability

The data used to support the findings of this study are available from the corresponding author upon request.

## References

[1] Siegel R., Naishadham D., Jemal A. (2012). Cancer statistics. *CA: A Cancer Journal for Clinicians*.

[2] Wallerek S., Sørensen J. B. (2015). Biomarkers for efficacy of adjuvant chemotherapy following complete resection in NSCLC stages I-IIIA. *European Respiratory Review*.

[3] Jiang H., Shao W., Zhao W. (2014). VEGF-C in non-small cell lung cancer: meta-analysis. *Clinica Chimica Acta*.

[4] Martin B., Paesmans M., Mascaux C. (2004). Ki-67 expression and patients survival in lung cancer: systematic review of the literature with meta-analysis. *British Journal of Cancer*.

[5] Tsao M. S., Aviel-Ronen S., Ding K. (2007). Prognostic and predictive importance of p53 and RAS for adjuvant chemotherapy in non small-cell lung cancer. *Journal of Clinical Oncology*.

[6] Yang S. L., Ren Q. G., Wen L., Hu J. L. (2016). Clinicopathological and prognostic significance of hypoxia-inducible factor-1 alpha in lung cancer: a systematic review with meta-analysis. *Journal of Huazhong University of Science and Technology*.

[7] Ren W., Mi D., Yang K. (2013). The expression of hypoxia-inducible factor-1α and its clinical significance in lung cancer: a systematic review and meta-analysis. *Swiss Medical Weekly*.

[8] Adamson R. T. (2013). Biomarkers and molecular profiling in non-small cell lung cancer: an expanding role and its managed care implications. *American Journal of Managed Care*.

[9] Li X., Liu X., Cui D., Wu X., Qian R. (2015). Clinical significance of nucleostemin and proliferating cell nuclear antigen protein expression in non-small cell lung cancer. *Journal of B.U.ON.: Official Journal of the Balkan Union of Oncology*.

[10] Singhal S., Vachani A., Antin-Ozerkis D., Kaiser L. R., Albelda S. M. (2005). Prognostic implications of cell cycle, apoptosis, and angiogenesis biomarkers in non-small cell lung cancer: a review. *Clinical Cancer Research*.

[11] Cherk M. H., Foo S. S., Poon A. M. (2006). Lack of correlation of hypoxic cell fraction and angiogenesis with glucose metabolic rate in non-small cell lung cancer assessed by 18F-fluoromisonidazole and 18F-FDG PET. *Journal of Nuclear Medicine*.

[12] Zhang J., Cui L. B., Tang X. (2014). DW MRI at 3.0 T versus FDG PET/CT for detection of malignant pulmonary tumors. *International Journal of Cancer*.

[13] Apostolova I., Ego K., Steffen I. G. (2016). The asphericity of the metabolic tumour volume in NSCLC: correlation with histopathology and molecular markers. *European Journal of Nuclear Medicine and Molecular Imaging*.

[14] Buck A. K., Halter G., Schirrmeister H. (2003). Imaging proliferation in lung tumors with PET: 18F-FLT versus 18F-FDG. *Journal of Nuclear Medicine*.

[15] Del Gobbo A., Pellegrinelli A., Gaudioso G. (2016). Analysis of NSCLC tumour heterogeneity, proliferative and 18F-FDG PET indices reveals Ki67 prognostic role in adenocarcinomas. *Histopathology*.

[16] Han B., Lin S., Yu L. J., Wang R. Z., Wang Y. Y. (2009). Correlation of ^18^F-FDG PET activity with expressions of survivin, Ki67, and CD34 in non-small-cell lung cancer. *Nuclear Medicine Communications*.

[17] Kaida H., Kawahara A., Hayakawa M. (2014). The difference in relationship between ^18^F-FDG uptake and clinicopathological factors on thyroid, esophageal, and lung cancers. *Nuclear Medicine Communications*.

[18] Kaira K., Oriuchi N., Shimizu K. (2009). Correlation of angiogenesis with ^18^F-FMT and ^18^F-FDG uptake in non-small cell lung cancer. *Cancer Science*.

[19] Kuyumcu S., Adalet I., Sanli Y., Turkmen C., Ozkan Z. G., Yilmazbayhan D. (2012). Somatostatin receptor scintigraphy with 111In-octreotide in pulmonary carcinoid tumours correlated with pathological and 18FDG PET/CT findings. *Annals of Nuclear Medicine*.

[20] Liu L. P., Zhang X. X., Cui L. B. (2017). Preliminary comparison of diffusion-weighted MRI and PET/CT in predicting histological type and malignancy of lung cancer. *Clinical Respiratory Journal*.

[21] Murakami S., Saito H., Sakuma Y. (2010). Correlation of 18F-fluorodeoxyglucose uptake on positron emission tomography with Ki-67 index and pathological invasive area in lung adenocarcinomas 30 mm or less in size. *European Journal of Radiology*.

[22] Nakamura H., Hirata T., Kitamura H., Nishikawa J. (2009). Correlation of the standardized uptake value in FDG-PET with the expression level of cell-cycle-related molecular biomarkers in resected non-small cell lung cancers. *Annals of Thoracic and Cardiovascular Surgery*.

[23] Nguyen X. C., Lee W. W., Chung J. H. (2007). FDG uptake, glucose transporter type 1, and Ki-67 expressions in non-small-cell lung cancer: correlations and prognostic values. *European Journal of Radiology*.

[24] Park S., Lee E., Rhee S. (2016). Correlation between semi-quantitative (18)F-FDG PET/CT parameters and Ki-67 expression in small cell lung cancer. *Nuclear Medicine and Molecular Imaging*.

[25] Sauter A. W., Winterstein S., Spira D. (2012). Multifunctional profiling of non-small cell lung cancer using 18F-FDG PET/CT and volume perfusion CT.. *Journal of Nuclear Medicine*.

[26] Shibata H., Nomori H., Uno K. (2009). 11C-acetate for positron emission tomography imaging of clinical stage IA lung adenocarcinoma: comparison with ^18^F-fluorodeoxyglucose for imaging and evaluation of tumor aggressiveness. *Annals of Nuclear Medicine*.

[27] Soussan M., Cyrta J., Pouliquen C. (2014). Fluorine 18 fluorodeoxyglucose PET/CT volume-based indices in locally advanced non-small cell lung cancer: prediction of residual viable tumor after induction chemotherapy. *Radiology*.

[28] Vesselle H., Salskov A., Turcotte E. (2008). Relationship between non-small cell lung cancer FDG uptake at PET, tumor histology, and Ki-67 proliferation index. *Journal of Thoracic Oncology*.

[29] Vesselle H., Schmidt R. A., Pugsley J. M. (2000). Lung cancer proliferation correlates with [F-18]fluorodeoxyglucose uptake by positron emission tomography. *Clinical Cancer Research*.

[30] Wang F. L., Tan Y. Y., Gu X. M. (2016). Comparison of positron emission tomography using 2-[18F]-fluoro-2-deoxy-D-glucose and 3-deoxy-3-[18F]-fluorothymidine in lung cancer imaging. *Chinese Medical Journal*.

[31] Watanabe K., Nomori H., Ohtsuka T. (2006). [F-18]Fluorodeoxyglucose positron emission tomography can predict pathological tumor stage and proliferative activity determined by Ki-67 in clinical stage IA lung adenocarcinomas. *Japanese Journal of Clinical Oncology*.

[32] Yamamoto Y., Nishiyama Y., Ishikawa S. (2007). Correlation of ^18^F-FLT and ^18^F-FDG uptake on PET with Ki-67 immunohistochemistry in non-small cell lung cancer. *European Journal of Nuclear Medicine and Molecular Imaging*.

[33] Yap C. S., Czernin J, Fishbein M. C. (2006). Evaluation of thoracic tumors with ^18^F-fluorothymidine and ^18^F-fluorodeoxyglucose-positron emission tomography. *Chest*.

[34] Taylor M. D., Smith P. W., Brix W. K. (2009). Fluorodeoxyglucose positron emission tomography and tumor marker expression in non-small cell lung cancer. *Journal of Thoracic and Cardiovascular Surgery*.

[35] Yang W., Zhang Y., Fu Z. (2010). Imaging of proliferation with ^18^F-FLT PET/CT versus ^18^F-FDG PET/CT in non-small-cell lung cancer. *European J Nuclear Medicine and Molecular Imaging*.

[36] Furukawa T., Miyata Y., Kushitani K. (2015). Association between [^18^F]-fluoro-2-deoxyglucose uptake and expressions of hypoxia-induced factor 1α and glucose transporter 1 in non-small cell lung cancer. *Japanese Journal of Clinical Oncology*.

[37] Kaira K., Endo M., Abe M. (2011). Biologic correlates of ^18^F-FDG uptake on PET in pulmonary pleomorphic carcinoma. *Lung Cancer*.

[38] Kaira K., Serizawa M., Koh Y. (2014). Biological significance of 18F-FDG uptake on PET in patients with non-small-cell lung cancer. *Lung Cancer*.

[39] Xing N., Cai Z. L., Zhao S. H., Yang L., Xu B. X., Wang F. L. (2011). The use of CT perfusion to determine microvessel density in lung cancer: comparison with FDG-PET and pathology. *Chinese Journal of Cancer Research*.

[40] Zhang Z. J., Chen J. H., Meng L. (2007). ^18^F-FDG uptake as a biologic factor predicting outcome in patients with resected non-small-cell lung cancer. *Chinese Medical Journal*.

[41] Araz O., Demirci E., Ucar E. Y. (2014). Roles of Ki-67, p53, transforming growth factor-β and lysyl oxidase in the metastasis of lung cancer. *Respirology*.

[42] Bai L., Guo C., Wang J. (2016). ^18^F-fludrodeoxyglucose maximal standardized uptake value and metabolic tumor burden are associated with major chemotherapy-related tumor markers in NSCLC patients. *OncoTargets and Therapy*.

[43] Duan X. Y., Wang W., Wang J. S., Shang J, Gao J. G., Guo Y. M. (2013). Fluorodeoxyglucose positron emission tomography and chemotherapy-related tumor marker expression in non-small cell lung cancer. *BMC Cancer*.

[44] Liu X., Zhang H., Yu X. (2014). The correlation of expression of VEGF and EGFR with SUV of (18)FDG-PET-CT in non-small cell lung cancer. *Contemporary Oncology*.

[45] Higashi K., Ueda Y., Sakurai A. (2000). Correlation of Glut-1 glucose transporter expression with [(18)F]FDG uptake in non-small cell lung cancer. *European Journal of Nuclear Medicine*.

[46] Khandani A. H., Whitney K. D., Keller S. M., Isasi C. R., Donald Blaufox M. (2007). Sensitivity of FDG PET, GLUT1 expression and proliferative index in bronchioloalveolar lung cancer. *Nuclear Medicine Communications*.

[47] Mamede M., Higashi T., Kitaichi M. (2005). [18F]FDG uptake and PCNA, Glut-1, and Hexokinase-II expressions in cancers and inflammatory lesions of the lung. *Neoplasia*.

[48] Takada K., Toyokawa G., Okamoto T. (2017). Metabolic characteristics of programmed cell death-ligand 1-expressing lung cancer on ^18^F-fluorodeoxyglucose positron emission tomography/computed tomography. *Cancer Medicine*.

[49] Zhang M., Wang D., Sun Q. (2017). Prognostic significance of PD-L1 expression and ^18^F-FDG PET/CT in surgical pulmonary squamous cell carcinoma. *Oncotarget*.

[50] Lopci E., Toschi L., Grizzi F. (2016). Correlation of metabolic information on FDG-PET with tissue expression of immune markers in patients with non-small cell lung cancer (NSCLC) who are candidates for upfront surgery. *European Journal of Nuclear Medicine and Molecular Imaging*.

[51] Surov A., Meyer H. J., Wienke A. (2018). Can imaging parameters provide information regarding histopathology in head and neck squamous cell carcinoma? A meta-analysis. *Translational Oncology*.

[52] Surov A., Meyer H. J., Wienke A. (2017). Correlation between apparent diffusion coefficient (ADC) and KI 67 in different tumors: a meta-analysis. Part 1: ADC_mean_. *Oncotarget*.

[53] Surov A., Meyer H. J., Wienke A. (2017). Correlation between apparent diffusion coefficient (ADC) and cellularity is different in several tumors: a meta-analysis. *Oncotarget*.

[54] Moher D., Liberati A., Tetzlaff J., Altman D. G. (2009). Preferred reporting items for systematic reviews and meta-analyses: the PRISMA statement. *PLoS Medicine*.

[55] Whiting P., Rutjes A. W., Reitsma J. B., Bossuyt P. M., Kleijnen J. (2003). The development of QUADAS: a tool for the quality assessment of studies of diagnostic accuracy included in systematic reviews. *BMC Medical Research Methodology*.

[56] Chalkidou A., Landau D. B., Odell E. W., Cornelius V. R., O’Doherty M. J., Marsden P. K. (2012). Correlation between Ki-67 immunohistochemistry and ^18^F-fluorothymidine uptake in patients with cancer: a systematic review and meta-analysis. *European Journal of Cancer*.

[57] Leeflang M. M., Deeks J. J., Gatsonis C., Bossuyt P. M. (2008). Systematic reviews of diagnostic test accuracy. *Annals of Internal Medicine*.

[58] Zamora J., Abraira V., Muriel A., Khan K., Coomarasamy A. (2006). Meta-DiSc: a software for meta-analysis of test accuracy data. *BMC Medical Research Methodology*.

[59] DerSimonian R., Laird N. (1986). Meta-analysis in clinical trials. *Controlled Clinical Trials*.

[60] Scimeca M., Urbano N., Bonfiglio R., Schillaci O., Bonanno E. (2018). Management of oncological patients in the digital era: anatomic pathology and nuclear medicine teamwork. *Future Oncology*.

[61] Schlüter C., Duchrow M., Wohlenberg C. (1993). The cell proliferation-associated antigen of antibody Ki-67: a very large, ubiquitous nuclear protein with numerous repeated elements, representing a new kind of cell cycle-maintaining proteins. *Journal of Cell Biology*.

[62] Deng S. M., Zhang W., Zhang B., Chen Y. Y., Li J. H., Wu Y. W. (2015). Correlation between the uptake of ^18^F-fluorodeoxyglucose (18F-FDG) and the expression of proliferation-associated antigen Ki-67 in cancer patients: a meta-analysis. *PLoS One*.

[63] Shen G., Ma H., Pang F., Ren P., Kuang A. (2018). Correlations of ^18^F-FDG and ^18^F-FLT uptake on PET with Ki-67 expression in patients with lung cancer: a meta-analysis. *Acta Radiologica*.

[64] Sholl L. M. (2015). Biomarkers in lung adenocarcinoma: a decade of progress. *Archives of Pathology & Laboratory Medicine*.

[65] Sharma S. V., Bell D. W., Settleman J., Haber D. A. (2007). Epidermal growth factor receptor mutations in lung cancer. *Nature Reviews Cancer*.

[66] Tateishi M., Ishida T., Mitsudomi T., Kaneko S., Sugimachi K. (1990). Immunohistochemical evidence of autocrine growth factors in adenocarcinoma of the human lung. *Cancer Research*.

[67] Inamura K. (2018). Update on immunohistochemistry for the diagnosis of lung cancer. *Cancers*.

[68] Tumeh P. C., Harview C. L., Yearley J. H. (2014). PD-1 blockade induces responses by inhibiting adaptive immune resistance. *Nature*.

[69] Simon S., Labarriere N. (2017). PD-1 expression on tumor-specific T cells: friend or foe for immunotherapy?. *Oncoimmunology*.

[70] Brody R., Zhang Y., Ballas M. (2017). PD-L1 expression in advanced NSCLC: insights into risk stratification and treatment selection from a systematic literature review. *Lung Cancer*.

[71] Gautschi O., Ratschiller D., Gugger M., Betticher D. C., Heighway J. (2007). Cyclin D1 in non-small cell lung cancer: a key driver of malignant transformation. *Lung Cancer*.

[72] Zhang L. Q., Jiang F., Xu L. (2012). The role of cyclin D1 expression and patient’s survival in non-small-cell lung cancer: a systematic review with meta-analysis. *Clinical Lung Cancer*.

[73] Villaflor V. M., Salgia R. (2013). Targeted agents in non-small cell lung cancer therapy: what is there on the horizon?. *Journal of Carcinogenesis*.

[74] Frezzetti D., Gallo M., Maiello M. R. (2017). VEGF as a potential target in lung cancer. *Expert Opinion on Therapeutic Targets*.

[75] Sharma A., Mohan A., Bhalla A. S. (2018). Role of various metabolic parameters derived from baseline ^18^F-FDG PET/CT as prognostic markers in non-small cell lung cancer patients undergoing platinum-based chemotherapy. *Clinical Nuclear Medicine*.

[76] Hong J. H., Kim H. H., Han E. J. (2016). Total lesion glycolysis using ^18^F-FDG PET/CT as a prognostic factor for locally advanced esophageal cancer. *Journal of Korean Medical Science*.

[77] Liao S., Penney B. C., Wroblewski K. (2012). Prognostic value of metabolic tumor burden on ^18^F-FDG PET in nonsurgical patients with non-small cell lung cancer. *European Journal of Nuclear Medicine and Molecular Imaging*.

[78] Lee H. Y., Hyun S. H., Lee K. S. (2010). Volume-based parameter of ^18^F-FDG PET/CT in malignant pleural mesothelioma: prediction of therapeutic response and prognostic implications. *Annals of Surgical Oncology*.

[79] Chung H. H., Kwon H. W., Kang K. W. (2012). Prognostic value of preoperative metabolic tumor volume and total lesion glycolysis in patients with epithelial ovarian cancer. *Annals of Surgical Oncology*.

